# Accidents Due to the Employment of Antipyrine

**Published:** 1897-11

**Authors:** 


### Accidents Due to the Employment of Antipyrine.

The *Gazette hebdomadaire de medecine et de chirurgie* for September 26th contains an article on this subject in which the writer refers to a thesis by M. V. Clement which, he says, is particularly instructive. The author devotes considerable space to the nature of the accidents which follow the immoderate use of antipyrine, giving a detailed account of those pertaining to the skin, the viscera, the nervous system, and the circulation, from which the following practical conclusions are reached. 1. Antipyrine should never be prescribed for very old people, for subjects attacked with non-compensated cardiac lesions, or for those in an adynamic condition. 2. In influenza and erysipelas it should always be associated with quinine and, in convalescence, with strychnine or caffeine. 3. In arthritic subjects, who are nearly always dyspeptics, it should be associated with an alkali (sodium bicarbonate or sodium benzoate) and prescribed in solution. If it cannot be taken except in a capsule, the patient should drink a quarter or half a glass of Vichy immediately after taking the capsule. 4. In tuberculous subjects twelve grains at a time should not be exceeded, and the condition of defervescence should be carefully watched. It is well in this case to combine alcohol and antipyrine and give the latter in solution. 5. In diabetic subjects the association with alkilies is obligatory.

6. In children antipyrine may be administered without inconvenience even in amounts proportionately larger than in adults, provided it is given in divided doses. This tolerance depends as much upon the integrity of the renal function as upon the mode of administration, which should nearly always be by the solution.

The writer calls attention to the fact that antipyrine given in powder, sometimes even in solution, has a special effect upon the mucous membrame of the stomach, and that this may be avoided by employing hypodermic injections. An injection done aseptically never gives place even to the least cutaneous poisoning.

The treatment of these accidents consists, naturally, in suspending the use of the drug. For the cutaneous accidents simple measures are generally sufficient. If it is a serious case of poisoning, injections of ether and especially of caffeine should be resorted to; during convalescence alcohol, digitalis, strychnine, and small doses of quinine render great service.—*New York Medical Journal.*

